# The First 1000 Days of Life Factors Associated with “Childhood Asthma Symptoms”: Brisa Cohort, Brazil

**DOI:** 10.1038/s41598-017-16295-4

**Published:** 2017-11-22

**Authors:** Joelma Ximenes Prado Teixeira Nascimento, Cecilia Claudia Costa Ribeiro, Rosângela Fernandes Lucena Batista, Maria Teresa Seabra Soares de Britto Alves, Vanda Maria Ferreira Simões, Luana Lopes Padilha, Viviane Cunha Cardoso, Elcio Oliveira Vianna, Heloisa Bettiol, Marco Antonio Barbieri, Antônio Augusto Moura Da Silva

**Affiliations:** 1 Federal University of Maranhão, Public Health Department, São Luís Maranhão, Brazil; 2 Federal University of Maranhão, Dentistry Department, São Luís Maranhão, Brazil; 3 Faculty of Medicine of Ribeirão Preto, São Paulo University, Department of Puericulture and Pediatrics, Ribeirão Preto São Paulo, Brazil

## Abstract

This prospective study used data from the BRISA Cohort, São Luís, Brazil (n = 1140) and analyzed associations between environmental factors up to the first 1000 days of life and “Childhood Asthma Symptoms”. “Childhood Asthma Symptoms” was a latent variable based on the number of wheezing episodes, emergency care visit due to wheezing, diagnosis of asthma and diagnosis of rhinitis. A theoretical model that included prenatal factors (socioeconomic status, pregestational body mass index-BMI, soft drink and junk food consumption), birth factors (gestational age, smoking and diseases during pregnancy, birth weight and type of delivery), first year of life factors (breastfeeding, environmental aeroallergens and respiratory diseases) and BMI z-score in the second year of life, was analyzed by structural equation modeling. High pregestational BMI, high soft drink consumption, cesarean section without labor, chill in the first three months of life, carpeted floor and child’s exposure to tobacco were associated with higher values of “Childhood Asthma Symptoms”. In contrast, high birth weight, breastfeeding and infant’s age were associated with lower values of “Childhood Asthma Symptoms”. These findings support the hypothesis that environmental factors that are present before conception and up to the first 1000 days of life are associated with asthma.

## Introduction

Asthma is the most prevalent nontransmissible chronic disease during childhood and is characterized by recurrent episodes of wheezing and shortness of breath that vary in frequency and severity among individuals (www.who.int)^1^. In children, asthma is a complex syndrome^2^. To date, the mechanisms involved in the etiology of asthma have not been completely elucidated^3,4^. However, the first 1000 days of life appear to have a strong influence on foetal programming and epigenetic regulation, thereby increasing the predisposition of children to many chronic diseases, including asthma^5^.

Accumulating evidence has indicated that epigenetic mechanisms contribute to the aetiology of asthma in children, and asthma can start early in the intrauterine life^6^. In addition, maternal exposures such as smoking during pregnancy^7^ and a high maternal pregestational body mass index (BMI)^8^ have consistently been associated with childhood asthma. At birth, other factors such as caesarian section^9,10^, especially pre-labour caesarean section^10^, low birth weight (LBW)^11^, and preterm birth (PTB)^12^ are also associated with a higher risk of childhood asthma. In the postnatal period, environmental stimuli including child’s exposure to tobacco and respiratory diseases at first year^7,13,14^, childhood obesity or rapid weight gain in early life have also been associated with childhood asthma^12,13^. Conversely, exclusive breastfeeding has been identified as a protective factor against asthma^14^.

In studies that have investigated environmental factors associated with childhood asthma, a traditional multiple regression approach has frequently been used, analyzing asthma as a dichotomous variable^8–14^. However, asthma in preschool children is a condition of difficult diagnosis^2^. In the present study, “Childhood Asthma Symptoms” was a continuous latent variable, deduced from the observed correlations among four clinical indicators: wheezing episodes, medical emergency visit due to intense wheezing, medical diagnosis of asthma and medical diagnosis of rhinitis. These indicators individually do not measure asthma well, but by using them together measurement error is reduced^15^.

The association between early life factors and “Childhood Asthma Symptoms” involves complex relationships of multicausality and temporality between variables, which can be better evaluated through structural equation modeling (SEM). Through this method, it is also possible to test, direct and indirect effects (mediation). Even if well-known risk factors for asthma have been studied, the structural equation modeling (SEM) approach allowed us to explore simultaneously direct and indirect effects of different variables at different stages of the life cycle (prenatal, birth and early life factors) in association with “Childhood Asthma Symptoms”. In the present study we tested the hypothesis of the association between prenatal, birth and postnatal factors with “Childhood Asthma Symptoms” in the first 1000 days of life. Thus, the objective of this prospective study was to analyze environmental factors during the prenatal period and the first 1000 days of life associated with “Childhood Asthma Symptoms” by using SEM.

## Results

**Figure 1:**
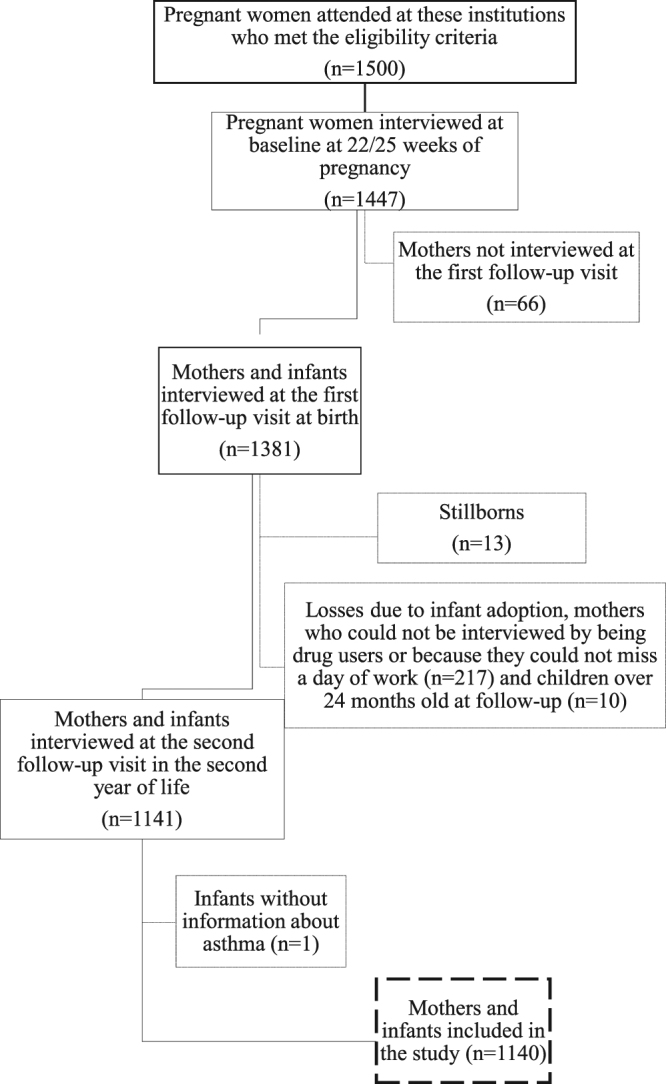
Flow diagram of the BRISA prenatal cohort, São Luís, Brazil.

The characteristics of the pregnant women and children of the São Luís BRISA cohort are listed in Tables 1 and 2. Figure 1 presents the flow diagram of the BRISA cohort (2010–2013).

**Table 1:** Sociodemographic and economic characteristics, life and nutritional habits, and reproductive health of women of the BRISA prenatal cohort, São Luís, Brazil, 2010–2013.

Variables	n	%
**Maternal schooling (years)**		
0–4	17	1.5
5–8	114	10.0
9–11	878	77.0
≥12	127	11.1
Missing*	4	0.4
**Occupation of the family head**		
Unskilled manual	308	27.0
Semiskilled manual	463	40.6
Skilled manual	51	4.5
Office functions	162	14.2
Higher level professional	58	5.1
Administrator/manager/director/owner	37	3.2
Missing*	61	5.4
**Family income (minimum wages)** ^**b**^		
<1	12	1.1
1 and<3	527	46.2
3 and<5	362	31.8
≥5	208	18.2
Missing*	31	2.7
**Economic class** ^**c**^		
D-E (poorest)	172	15.1
C	746	65.4
A-B (wealthiest)	170	14.9
Missing*	52	4.6
**Soft drink consumption (tertiles)**		
1^st^ (no consumption)	492	43.2
2^nd^ (once a week)	284	24.9
3^rd^ (two or more times a week)	357	31.3
Missing*	7	0.6
**Junk food consumption (tertiles)**		
1^st^ (up to once a week)	544	47.7
2^nd^ (once or twice per week)	336	29.5
3^rd^ (two or more times a week)	259	22.7
Missing*	1	0.1
**Smoking status during the current pregnancy**		
Non-smoker	1109	97.3
Smoker	28	2.5
Missing*	3	0.2
**Hypertension during the current pregnancy**		
No	948	83.2
Yes	189	16.6
Missing*	3	0.2
**Diabetes during the current pregnancy**		
No	1103	96.8
Yes	33	2.9
Missing*	4	0.3
**Respiratory diseases during the current pregnancy**		
No	1096	96.2
Yes	39	3.4
Missing*	5	0.4
**Type of delivery**		
Vaginal	566	49.6
Caesarean section with labour	318	27.9
Caesarean section without labour	253	22.2
Missing*	3	0.3
**Gestational age (weeks)**		
Preterm (<37)	59	5.1
Early term (37–38)	222	19.5
Full term and late term (39–41)	792	69.5
Post-term (≥42)	64	5.6
Missing*	3	0.3
**Total**	**1140**	**100.0**

^a^The SES variable latent for the São Luís BRISA cohort was validated in a previous study^45^. ^b^Monthly family income based on the monthly Brazilian minimum wage (approximately US$ 290.00 in 2010), categorized as: <1, >1 and <3, 3– and <5, and ≥5. ^c^Economic class according to the Criteria of Economic Classification – Brazil^30^. *Values not available. Mean maternal age: 26.12 years/standard deviation ± 5.58. Mean maternal pregestational BMI: 23.08 kg/m^2^/standard deviation ± 3.98.

**Table 2:** Demographic, nutritional, and health characteristics of the children of the BRISA prenatal cohort, São Luís, Brazil, 2010–2013.

Variables	n	%
**Sex**		
Male	571	50.1
Female	564	49.5
Missing*	5	0.4
**Skin color**		
White	312	27.4
Others **	746	65.4
Black	80	7.0
Missing*	2	0.2
**Exclusive breastfeeding (months)**		
<6	503	44.1
≥6	616	54.1
Missing*	21	1.8
**Chill in the first three months of life**		
No	834	73.2
Yes	302	26.5
Missing*	4	0.3
**Hospitalization due to respiratory tract problems up to the first year of life**		
No	948	83.2
Yes	77	6.8
Missing*	115	10.0
**Medical diagnosis of asthma**		
No	1108	97.2
Yes	32	2.8
**Number of wheezing episodes**		
0	802	70.3
<3	256	22.5
3–6	52	4.6
>6	20	1.8
Missing*	10	0.8
**Medical emergency care visit due to intense wheezing**		
No	976	85.6
Yes	159	13.9
Missing*	5	0.5
**Medical diagnosis of rhinitis**		
No	1062	93.2
Yes	73	6.4
Missing*	5	0.4
**Presence of pet at home**		
No	645	56.6
Yes	486	42.6
Missing*	9	0.8
**Carpet covered floor**		
No	953	83.6
Yes	183	15.1
Missing*	4	0.3
**Exposure to mold/mildew**		
No	968	84.9
Yes	162	14.2
Missing*	10	0.9
**Child**’**s exposure to tobacco**		
No	791	69.4
Yes	193	16.9
Missing*	156	13.7
**Total**	**1140**	**100.00**

*Values not available. **Included: mulatto/half-breed/brown (miscegenation of black and white Brazilians) plus a minority of oriental (n = 12). Mean infant’s age: 15.89 months/standard deviation ± 2.06. Mean infant’s birth weight: 3.27 grams/standard deviation ± 0.48. Mean child’s BMI z-score: 0.62/standard deviation ± 1.27.

The confirmatory factor analysis (CFA) model that tested the “Childhood Asthma Symptoms” construct validity showed a good fit. A pathway starting from wheezing and progressing towards medical emergency care visit due to intense wheezing (modification index 61.905) was incorporated into the construct and later analyzed by SEM. Validation results for the “Childhood Asthma Symptoms” latent variable are listed in Table 3.

**Table 3:** Fit indices for the initial and final confirmatory factor analysis models and the initial and final structural equation models. São Luís, Brazil, 2010–2013.

Indices model fit	Initial CFA^a^	Final CFA^b^	Initial SEM model^c^	Final SEM model^d^
*X* ^2e^	10.541	0.471	411.490	432.926
Degrees of freedom	2	1	164	318
*p-value X* ^2^	0.0051	0.4926	<0.001	<0.001
RMSEA^f^	0.061	<0.001	0.036	0.018
90% CI^g^	0.028–0.099	0.000–0.069	0.032–0.041	0.013–0.022
*p*-value	0.253	0.851	1.000	1.000
CFI^h^	0.995	1.000	0.928	0.960
TLI^i^	0.985	1.002	0.889	0.951
SRMR^j^	0.692	0.089	—	—
WRSM^l^	—	—	1.147	0.914

^a^Confirmatory factor analysis (CFA) of the “Childhood Asthma Symptoms”. ^b^CFA of the “Childhood Asthma Symptoms”. ^c^Initial theoretical model proposed. ^d^Final model analyzed by SEM. ^e^Chi-square test. ^f^RMSEA: root mean square error of approximation. ^g^CI: confidence interval. ^h^CFI: comparative fit index. ^i^TLI: Tucker Lewis index. ^j^SRMR: standardized root mean square residual. ^l^WRSM: weighted root mean square residual.

The initial proposed theoretical model did not show a good fit according to the Tucker-Lewis Index - TLI indicator (0.889), which would be desirable to be greater than >0.95^15^; and also according to the Weighted Root Mean Square Residual - WRMR indicator (1.147), which ideally should be lower than one (<1)^15^. Then, we followed the modification index suggestion. The higher modification index suggested (value = 108.879) was to include a pathway starting from maternal age towards the junk food variable. This modification originated the final model, which had a good fit according to the TLI indicator (0.951) and the WRMR indicator (0.914) (Table 3). In the final model, each indicator of the Socioeconomic Status (SES) and the “Childhood Asthma Symptoms” constructs had factor loadings greater than 0.5 and all the indicators of these constructs had significant p-values (Table 4).

**Table 4:** Factor loadings, standard errors and p-values of the indicators of the latent variables Socioeconomic Status and “Childhood Asthma Symptoms”, São Luís, Brazil, 2010–2013.

Latent variable	Factor loadings	Standard error	*p-value*
**Socioeconomic Status (SES)**			
Family income	0.727	0.031	<0.001
Maternal schooling (years)	0.543	0.035	<0.001
Occupation of the family head	0.548	0.030	<0.001
Economic class	0.793	0.032	<0.001
**“Childhood Asthma Symptoms”**			
Number of wheezing episodes	0.545	0.062	<0.001
Medical emergency visit due to intense wheezing	0.792	0.067	<0.001
Medical diagnosis of asthma	0.733	0.085	<0.001
Medical diagnosis of rhinitis	0.624	0.074	<0.001

A higher pregestational BMI (Standardized Coefficient – SC = 0.121; p = 0.032), caesarean section without labour (SC = 0.136; p = 0.046), high soft drink consumption during pregnancy (SC = 0.122; p = 0.044), chill in the first three months of life (SC = 0.311; p < 0.001), carpet covered floor (SC = 0.142; p = 0.023) and child’s exposure to tobacco (SC = 0.198; p = 0.027) were associated with higher values of “Childhood Asthma Symptoms” (Table 5).

**Table 5:** Standardized coefficients, standard errors, and p-values for the total and direct effects of the structural equation model up to the first 1000 days of life factors and “Childhood Asthma Symptoms”. São Luís, Brazil, 2010–2013.

“Childhood Asthma Symptoms”^a^	Total Effects			Direct Effects		
	Standardized coefficient	Standard error	p-value	Standardized coefficient	Standard error	p-value
Socioeconomic Status	−0.035	0.063	0.573	−0.030	0.077	0.695
Mother’s age at delivery	0.042	0.052	0.419	0.030	0.075	0.687
Soft drink consumption	**0.122**	**0.061**	**0.044**	**0.137**	**0.062**	**0.028**
Junk food intake	0.022	0.063	0.729	−0.011	0.079	0.893
Pregestational BMI	**0.121**	**0.057**	**0.032**	**0.127**	**0.062**	**0.041**
Maternal smoking during pregnancy	0.059	0.131	0.653	0.049	0.165	0. 767
Hypertension during gestation	−0.075	0.095	0.429	0.001	0.101	0.995
Gestational diabetes	−0.002	0.148	0.987	−0.034	0.168	0.837
Respiratory diseases during pregnancy	0.096	0.145	0.510	0.096	0.145	0.510
Type of delivery	**0.136**	**0.068**	**0.046**	**0.146**	**0.070**	**0.038**
Gestational age (weeks)	−0.095	0.070	0.174	0.020	0.103	0.848
Birth weight	−**0.207**	**0.094**	**0.028**	−**0.210**	**0.093**	**0.024**
Exclusive breastfeeding for six months	−**0.134**	**0.065**	**0.038**	−0.099	0.064	0.126
Chill in the first three months of life	**0.311**	**0.066**	<**0.001**	**0.311**	**0.066**	<**0.001**
Presence of pet at home	−0.081	0.064	0.206	−0.081	0.064	0.206
Carpet covered floor	**0.142**	**0.063**	**0.023**	**0.142**	**0.063**	**0.023**
Exposure to mold/mildew	0.054	0.077	0.482	0.054	0.077	0.482
Child’s exposure to tobacco	**0.198**	**0.090**	**0.027**	**0.203**	**0.093**	**0.030**
Child’s BMI z-score	0.032	0.065	0.625	0.032	0.065	0.625
Infant’s age	−**0.141**	**0.051**	**0.006**	−**0.143**	**0.052**	**0.006**
Skin color	−0.070	0.051	0.173	−0.070	0.051	0.173
Sex	−0.081	0.052	0.119	−0.082	0.052	0.115

BMI: body mass index. ^a^The latent variable “Childhood Asthma Symptoms” was defined by the number of wheezing episodes, emergency care visit due to intense wheezing, medical diagnosis of asthma, and medical diagnosis of rhinitis.

Exclusive breastfeeding for six months also had a negative total effect (SC = −0.134; p = 0.038) (Table 5); and also an indirect effect via reducing chill in the first three months of life (SC = −0.035; p = 0.047) resulting in lower “Childhood Asthma Symptoms” values.

Gestational age also had a negative indirect effect on the “Childhood Asthma Symptoms”, with this effect being mediated by birth weight (SC = −0.111; p = 0.025).

Hospitalization due to respiratory tract problems up to the first year of life (otitis, tonsillitis, pneumonia and pharyngitis) was correlated to higher values of “Childhood Asthma Symptoms” (SC = 0.689; p < 0.001).

A correlation was also observed between maternal smoking during pregnancy and child’s exposure to tobacco (SC = 0.290; p < 0.001).

## Discussion

The continuous latent variable “Childhood Asthma Symptoms” was based on four indicators, and all had factor loadings greater than 0.5. The CFA model showed good fit indices, indicating that this construct adequately represents what it proposes to measure. Here we used a latent variable, a variable that was derived indirectly based on the observed correlations between the variables number of wheezing episodes, emergency care visit due to intense wheezing, medical diagnosis of asthma and medical diagnosis of rhinitis, to measure “Childhood Asthma Symptoms”. By using a latent variable, it is possible to reduce measurement error^15,16^. Because asthma is difficult to measure, we used “Childhood Asthma Symptoms” based on symptoms commonly associated with asthma as a latent variable^2^. By using a latent variable, it was also possible to reduce the probability of type II error (false negative) in the associations between the first 1000 days of life factors and “Childhood Asthma Symptoms”^15^.

The “Childhood Asthma Symptoms” construct also included indicators of asthma that encompassed allergic rhinitis in children, as suggested by the orientation of the convergent loadings observed in the exploratory factor analysis (EFA) and the CFA. These data support the hypothesis that a “united allergic airway”^17^ is involved in these two diseases. Correspondingly, epidemiological evidence has shown a strong relationship between allergic rhinitis and asthma^17,18^ thereby suggesting that these two conditions share common physiopathology aspects^18^.

A high pregestational BMI, high soft drink consumption during pregnancy, cesarean section without labor, chill in the first three months of life, carpet covered floor and child’s exposure to tobacco were associated with higher “Childhood Asthma Symptoms” values. In contrast, high birth weight and infant’s age were associated with lower “Childhood Asthma Symptoms” values. Exclusive breastfeeding for six months had a negative effect on “Childhood Asthma Symptoms” values, and also an indirect effect via reducing chill in the first three months of life.

A higher maternal pregestational BMI value was one of the factors identified as being associated with increased values of “Childhood Asthma Symptoms”. This result supports the data of a meta-analysis that showed an association between elevated pregestational BMI and a high risk of childhood asthma/wheezing^8^. Birth by caesarean section without labour was also associated with “Childhood Asthma Symptoms” in the present study. In other studies that used standard multiple regression models, a positive association between caesarean section delivery, especially caesarean section without labour^10^, and childhood asthma was observed^8–14^. Various mechanisms have been proposed to explain this association: a) colonization of a baby’s intestinal tract with bacteria may be affected by a caesarean delivery and this could influence the future risk of childhood asthma^9^; b) the absence of a hormonal response to delivery involving cortisol and catecholamine among other hormones could lead to a reduction in the immunological response of infants, thereby increasing the future risk of asthma^10^; and c) caesarean sections could potentially result in a higher risk of respiratory distress syndrome and transitory tachypnea in neonates^19^, as these are both risk factors for asthma^20^.

Meta-analysis results have also shown that preterm birth significantly increases the risk of childhood asthma^12^. While traditional multiple regression models were previously employed, our data was analyzed by SEM. In our study, a higher GA was indirectly associated with lower “Childhood Asthma Symptoms” values, which was almost completely mediated by birth weight. In addition, a higher birth weight was associated with lower “Childhood Asthma Symptoms” values. Therefore, the results of the present study support the findings of a previous meta-analysis in which low birth weight was found to significantly increase the risk of childhood asthma^11^.

The present data did not show an association between junk food consumption during pregnancy and “Childhood Asthma Symptoms”. However, a higher tertile of soft drink consumption during the prenatal period was associated with higher “Childhood Asthma Symptoms” values. The sugar added to soft drinks may explain this association. In a study of 53 countries, an association between per capita sugar consumption during the perinatal period and subsequent risk of severe asthma symptoms in children was observed^21^. It is also possible that non-sugar ingredients contributed to the observed association between soft drinks and “Childhood Asthma Symptoms” in the present study. In another cohort study, consumption of artificially sweetened soft drinks during pregnancy was associated with childhood asthma^22^.

It is possible that nutritional variables during the first 1000 days of life influence epigenetic regulatory mechanisms and this can lead to a predisposition towards many nontransmissible chronic diseases, including asthma^5^. For example, foetal exposure to certain feeding patterns has been shown to induce a proinflammatory milieu that has the potential to affect foetal immunity and pulmonary development, thereby affecting the onset of childhood asthma^8^.

Maternal infection variables were not associated with “Childhood Asthma Symptoms” in our study, possibly because serious infections in pregnancy were not frequently reported (only 3.4% of pregnant women - n = 39 - reported pneumonia) and other non-serious airways infections may have been underreported by pregnant women. However, regarding child’s respiratory infections, chill in the first three months of life was associated with higher “Childhood Asthma Symptoms” values and hospitalization due to respiratory tract problems up to the first year of life (pneumonia, otitis, tonsillitis, pharyngitis, influenza and sinusitis) was correlated to higher values of “Childhood Asthma Symptoms”. It is possible that some of the reports of wheezing are consequences of infectious conditions, for example, respiratory syncytial virus (RSV). It is well documented that RSV is the most common trigger of bronchiolitis and that rhinovirus is the biological agent which is mainly associated with childhood asthma^14^. Thus, children’s infections may even result in lifelong asthma.

In our study, post-birth environmental stimuli (carpet covered floor, pets and mold/mildew) were associated to higher “Childhood Asthma Symptoms” values. In agreement with our results, exposure to allergens in early childhood has been implicated in asthma pathogenesis in children^14^ and birth cohort results have also shown that carpet covered floor in the child’s bedroom is associated with greater prevalence of wheezing in childhood^23^.

Also in relation to post-birth environmental aeroallergens, our data showed that child’s exposure to tobacco was associated to higher “Childhood Asthma Symptoms” values. Passive exposure to smoking in the postnatal period has been showed to increase the risk of asthma in early chidhood^7^. However, different from what was expected^7^, maternal smoking during pregnancy was not associated with higher values of “Childhood Asthma Symptoms”. This could be explained by the lower maternal smoking rate in the present study (2.6%), compared to Brazilian national^24^ data (9.6%) and US population-data^25^ (12%); furthermore, a lower smoking rate has been showed in the city of São Luís compared to other Brazilian cities^26^. Additionally, our data also showed a correlation between maternal smoking during pregnancy and child’s exposure to tobacco.

Children who were exclusively breastfed for six months had lower “Childhood Asthma Symptoms” values, mediated by chill in the first three months of life. It is well established that infant breastfeeding is strongly associated with lower risks of respiratory illnesses in later childhood and adolescence. A systematic review showed that breastfeeding is a protective factor against childhood asthma^14^. Nursing provides exposure to maternal antibodies and favors the production of anti-inflammatory cytokines that appear to protect children against asthma^14^.

During the second year of life, no effect of BMI z-scores on “Childhood Asthma Symptoms” was observed. Previously, systematic reviews demonstrated that overweight or obesity appeared not to be associated with an increased risk of childhood asthma^13^. However, the causal pathway and temporal aspects of this relationship remain to be elucidated, and also warrant more in-depth epidemiological investigations^13^. Adjustment in our model for variables that precede childhood BMI (e.g., pregestational BMI, consumption of soft drinks during pregnancy, caesarian section without labour, and birth weight) may account for the absence of an observed association between BMI z-scores and “Childhood Asthma Symptoms” in contrast with the results of previous studies^12,13^.

There were three significant strengths of the current prospective study. One, data were collected at three distinct time points: during the intrauterine period, following birth, and at the second year of life. Thus, a temporal analysis of independent variables in regard to asthma was performed. Second, the latent variable “Childhood Asthma Symptoms” encompassed four indicators, and measurement errors in the determination of asthma were minimized. Third, SEM was used to evaluate the associations.

Among the limitations of the present study we point out the use of a convenience sample in the BRISA Prenatal Cohort, due to the difficulty of obtaining a random sample representative of the population of pregnant women in São Luís, Brazil. To analyze the representativeness of our data with relation to the general population, we compared the frequencies of some variables in our sample (the BRISA Prenatal Cohort) with the random sample of “the BRISA Birth Cohort”^27^, a population-based study that was conducted during the same period in the city of São Luís, by the chi-square test. We observed that only the intermediate categories of maternal schooling were different in the two cohorts (5 to 8 years of study and 9 to 11 years of study), showing that in our sample the pregnant women were slightly better educated compared to the population-based sample. However, the frequencies of the other variables, ie. smoking during pregnancy, gestational age and birth weight did not differ in the two cohorts, reinforcing the external validity of our data.

In the present study we did not perform allergy tests. Although they would be desirable and would allow better characterization of the sample with relation to the allergic status of the children, considerations about their costs due to the high sample size, and their accuracy in the first years of life^28^ weighed more on the choice.

## Conclusions

High pregestational BMI, high soft drink consumption during pregnancy, and cesarean section without labor, chill in the first three months of life, carpet covered floor, and child’s exposure to tobacco were factors associated with higher “Childhood Asthma Symptoms” values. In contrast, high birth weight, exclusive breastfeeding for six months and infant’s age were associated with lower “Childhood Asthma Symptoms” values. These findings highlight that asthma prevention for children might start before conception. Moreover, guidelines for healthy eating habits might be included in obstetrical practice along with an emphasis on the need to reduce caesarian section rates without medical indication and the importance of exclusive breastfeeding for six months for the prevention of asthma and respiratory diseases in the first year of life. The repercussions of these guidelines might not only improve the health of mother and child, but might also have a lifelong impact as a result of a reduced incidence of asthma.

## Methods

### Study design

This was a prospective cohort study entitled: “Etiological factors of preterm birth and consequences of perinatal factors on the child’s health: birth cohorts in two Brazilian cities, Ribeirão Preto and São Luís - BRISA”^27^. In this analysis we used data available from São Luís, a city located in the northeast region of Brazil^29^.

### Participants and sample

This prenatal cohort consisted of a convenience sample due to the difficulty in obtaining a random sample representative of the population of pregnant women in São Luís, Brazil. Thus, pregnant women attending prenatal outpatient clinics, public, or private hospitals of São Luís were invited to participate in this study. Inclusion criteria were: completion of a first ultrasound exam at less than 20 weeks of gestational age (GA) and an intent to give birth at one of the maternity hospitals in the municipality where the prenatal interview was held. Only women with singleton pregnancies were included.

### Data collection

A prenatal questionnaire was used to collect the following baseline information: maternal age (years), monthly family income (multiples of the minimum wage), maternal schooling (years of study), occupation of the family head, economic class according to the Criterion of Economic Classification Brazil (A-B being the wealthiest, C, D-E being the poorest)^30^, soft drink consumption, junk food intake, reported weight before pregnancy, and measured height^31^.

Soft drink consumption during pregnancy was determined based on two questions: 1) “On how many days of the week do you consume soft drinks?” and 2) “How many times a day do you ingest soft drinks?”. Junk food intake was determined based on the Block scoring system^32^ that considered the consumption of hamburgers, cheeseburgers, ham and cheese hot sandwiches, sausages, hot dogs, salame, ham, baloney, cold cuts, french fries, packaged snacks, and popcorn.

The initial birth questionnaire was reviewed at the first follow-up at birth to obtain the most accurate information regarding: maternal age (years), smoking during pregnancy, self-reported diabetes, hypertension based on medical diagnosis and respiratory diseases during pregnancy: bronchitis, asthma, bronchiolitis, wheezing, otitis, pneumonia, tonsillitis and pharyngitis, type of delivery (vaginal or caesarian), whether the mother had started labour with pain, GA-based obstetrical ultrasound (OU), date of last menstruation (DLM), and birth weight obtained from the neonate’s medical records.

At the second year of life follow-up, the following information was obtained: infant’s age (months), sex, skin color, respiratory symptoms related to “Childhood Asthma Symptoms”, status of exclusive breastfeeding, child’s respiratory infections during the first year of life, infant’s weight and height, environmental stimuli that included allergens (exposure to carpet covered floor, pets and mold/mildew) and child’s exposure to tobacco. Exclusive breastfeeding was defined according to the response obtained to the following question: “Up to what age was your baby exclusively breastfed, with the exclusion of tea, water, other types of milk, other drinks, and foods?”. Data regarding child’s respiratory infections during the first year of life were obtained by the following questions: chill in the first three months of life (cold, runny nose, sneezing, nasal obstruction, cough, with or without fever) and hospitalization due to respiratory tract problems up to the first year of life (pneumonia, otitis, tonsillitis, pharyngitis, influenza and sinusitis). The baby’s weight was measured with a digital scale with 0.1 kg precision (Tanita^®^, Arlington Heights, IL, USA). Height for mother and child was measured with a portable stadiometer with 0.1 cm precision (Alturexata^®^, Belo Horizonte, Minas Gerais, Brazil)^31,33^.

### Endogenous/dependent variables

Maternal age (years) was used as a discrete numerical variable in our model. In contrast, pregestational BMI^32^ was used as a continuous numerical variable. Soft drink consumption was obtained by multiplying the frequency of weekly intake (0–7×/week) by daily intake (1–6×/day) and categorized into tertiles. Junk food intake was evaluated as the sum of the food items previously described and then was categorized as <3×/month, 1–2×/week, or ≥5×/week. Smoking, hypertension, diabetes and respiratory diseases during the current pregnancy were self-reported and treated as dichotomous variables (yes or no).

Type of delivery was treated as a categorical variable (vaginal, caesarian with labour, and caesarian without labour), with the latter category referring to pregnant women who did not start labour with pain and underwent a caesarian section. GA was calculated based on two criteria: DLM or OU obtained at a GA <20 weeks. When GA was measured according to DLM and differed by ≤10 days compared with the value estimated by OU, GA was calculated according to the DLM; when the difference in GA was >10 days between the use of DLM versus OU, GA was estimated according to the OU^34^. GA was categorized as: preterm (<37 gestational weeks), early term (37–38 gestational weeks), full term and/or late term (39–41 gestational weeks), and post-term (≥42 gestational weeks)^35^.

Child’s sex was a dichotomous variable (male = 1 or female = 2). Skin color was reported by the child’s mother as white, oriental, miscegenation of black and white Brazilians (mullato/brown), or black. Infant’s age was used as a discrete numerical variable.

Birth weight and BMI z-score^36^ were treated as continuous numerical variables, while exclusive breastfeeding was treated as a dichotomous categorical variable: <6 months or ≥6 months.

Chill in the first three months of life, hospitalization due to respiratory tract problems up to the first year of life, environmental stimuli that included allergens (carpet covered floor, pets and mold/mildew), child’s exposure to tobacco were self-reported and treated as dichotomous variables (yes or no).

### Statistical analysis

Because of the occurrence of losses to follow-up, all variables were compared between the children who were seen for a second follow-up and those who were not, using the Chi-square test. Fewer children that were born at an earlier GA attended the second follow-up visit. On this basis, the sample was weighted by calculating the probability of a children being seen at the second follow-up visit as a function of GA using a logistic regression model. The inverse of this probability of selection was then calculated and this variable was used to weight the SEM estimates.

### “Childhood Asthma Symptoms” latent variable

The “Childhood Asthma Symptoms” latent variable consisted of four indicators: number of wheezing episodes, emergency care visit due to intense wheezing, medical diagnosis of asthma and medical diagnosis of rhinitis. The response options for each of these questions were dichotomous (no or yes), except for the number of wheezing episodes which were categorized as: 0, <3, 3–6, and >6 episodes. These indicators were selected based on the convergent loadings (>0.50) in an EFA. This latent variable had its construct validity evaluated later by CFA using the Mplus software (version 7.0). Model fit was assessed based on the following fit indices: a) a p-value < 0.05 in the Chi-square test (*χ* ^2^)^15^; b) p > 0.05 and an upper 90% confidence interval limit <0.08 for the Root Mean Square Error of Approximation (RMSEA); c) Comparative Fit Index (CFI) and Tucker-Lewis Index (TLI) values >0.95, and d) a WRMR value <1^15^. The modindices command was used to calculate the modification indices and thus to identify new paths that could improve the model fit^16^.

### SEM

**Figure 2:**
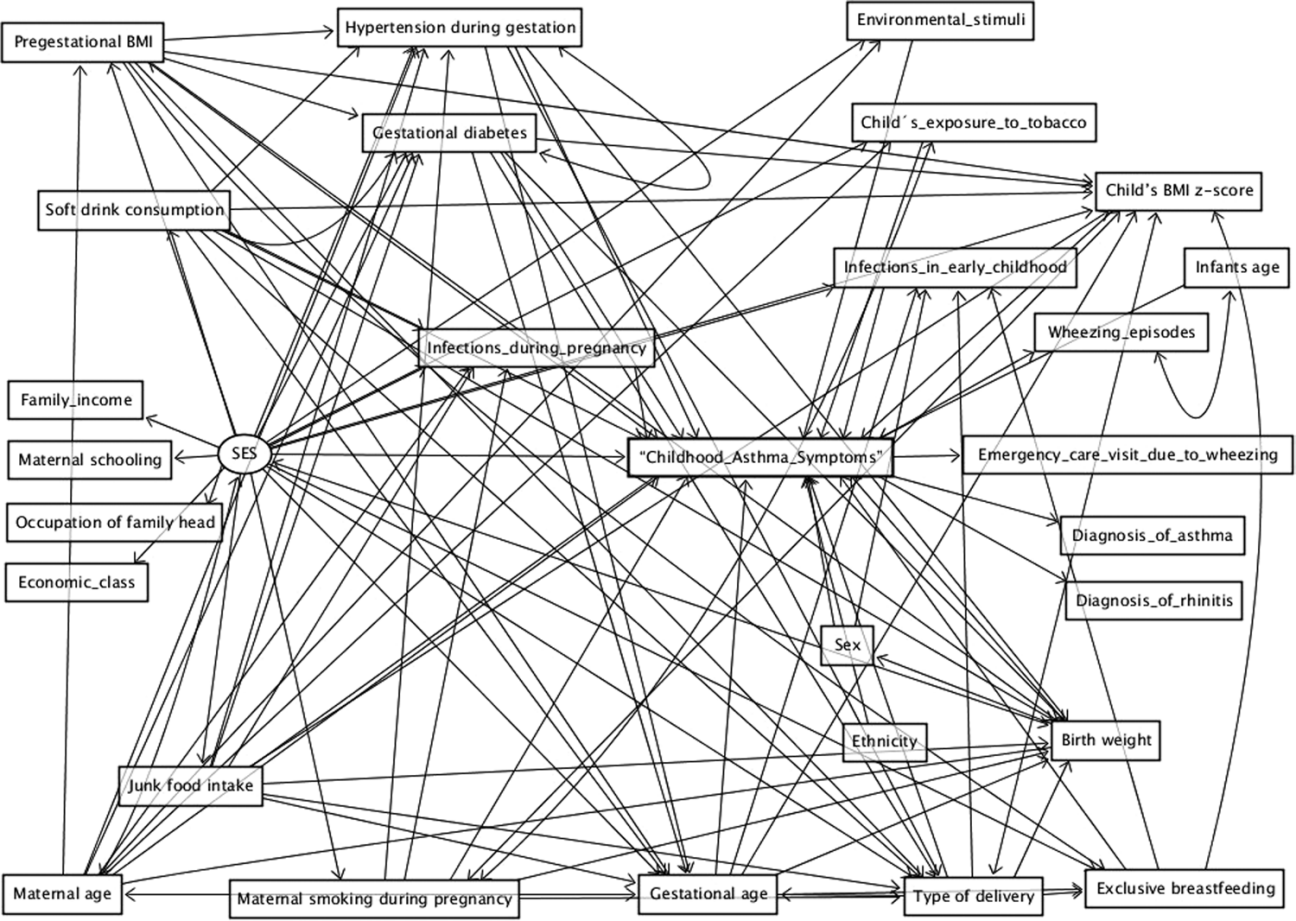
Proposed theoretical model for the environmental factors associated with “Childhood Asthma Symptoms” during the prenatal period and the first 1000 days of life: BRISA cohort, São Luís, Brazil, 2010–2013. The Socioeconomic Status (SES) would be a more distal determinant (exogenous variable) exerting its effects on the development of “Childhood Asthma Symptoms” in early childhood^38^ and on the remaining dependent variables of the model. Maternal age potentially affects pregestational body mass index (BMI)^39^ and “Childhood Asthma Symptoms”^8^. Maternal respiratory infections may be associated with “Childhood Asthma Symptoms”^14^. Smoking during pregnancy may result in “Childhood Asthma Symptoms”^7^ and may also result in low birth weight (LBW)^40^; soft drink and junk food consumption potentially affect blood pressure^41,42^ and diabetes^43^, which may lead to caesarean delivery, which has been associated with “Childhood Asthma Symptoms”^9,10^, LBW^11^ and preterm birth (PTB)^12,13^. Girls have a lower risk of “Childhood Asthma Symptoms” and black race is associated with an increased risk of “Childhood Asthma Symptoms”^3,44^. Exclusive breastfeeding may have a protective effect on “Childhood Asthma Symptoms”^14^. Child’s respiratory infections during the first year of life may be associated with “Childhood Asthma Symptoms”^14^, while higher child’s BMI z-score has been associated with “Childhood Asthma Symptoms”^12,13^. Environmental stimuli in early childhood (exposure to carpet covered floor, pets and mold/mildew), including exposure to tobacco have been associated with “Childhood Asthma Symptoms”^7,14,23^.

The Fig. 2 presents the proposed theoretical model to analyze the environmental factors associated with “Childhood Asthma Symptoms” during the prenatal period and the first 1000 days of life using SEM.

In the SEM the Weighted Least Squares Mean and Variance Adjusted (WLSMV) estimator and theta parameterization were used^37^. The same previously described fit indices^15,16^ were considered to determine whether the model showed good fit. The Chi-square, degrees of freedom, and p values were evaluated, but were not adopted as parameters to assess model fitting due to their sensitivity to sample size.

The modindices command was also used to test if a new path could improve model fit. When the modification index was >10 and the suggested modification were considered plausible from a theoretical standpoint a new model including that path was generated^16^. The total, direct and indirect effects were estimated.

### Ethical aspects

The Research Ethics Committee of the University Hospital of the Federal University of Maranhão, Brazil (protocol no. 4771/2008-30), approved this study. All methods were performed in accordance with relevant guidelines and regulations of that Research Ethics Committee. The written informed consent was obtained from all women and for those younger than 18 years an accompanying adult also signed the consent form. The confidentiality, image protection and non-stigmatization were guaranteed to all participants.

### Data availability

The datasets generated during and/or analyzed during the current study are available from the corresponding author on request.
